# Cardiac Arrest Secondary to Bilateral Pulmonary Emboli following Arteriovenous Fistula Thrombectomy: A Case Report with Review of the Literature

**DOI:** 10.1155/2012/831726

**Published:** 2012-05-23

**Authors:** Avni Shah, Naheed Ansari, Zaher Hamadeh

**Affiliations:** Renal Division, Department of Medicine, Jacobi Medical Center, Albert Einstein College of Medicine, 1400 Pelham Parkway South, Bronx, NY 10461, USA

## Abstract

Number of patients with End Stage Renal Disease (ESRD) is growing worldwide. Hemodialysis remains the main modality of renal replacement therapy for ESRD patients. A patent hemodialysis access (arteriovenous fistula or arteriovenous graft) plays a key role in successful delivery of hemodialysis. Common vascular access issues encountered by patients and nephrologists are thrombosis and infection. The thrombosed access is declotted by various percutaneous techniques these days by multiple outpatient access centers in a timely fashion. Thrombolysis can give rise to various complications, a few of which can be life threatening. A young hemodialysis patient underwent percutaneous thrombolysis of his clotted arteriovenous fistula. Outpatient access thrombectomy was complicated immediately afterwards with cardiac arrest requiring cardiac resuscitation in the recovery room. The patient was admitted to intensive care unit after life sustaining care. Work up revealed multiple pulmonary emboli to both lung fields on CT scan of the chest. Patient was anticoagulated and discharged from the hospital. Thrombolysis of clotted hemodialysis access is associated commonly with occurrences of pulmonary embolic which are usually asymptomatic. Massive pulmonary embolization due to access thrombolysis is rare. Nephrologists and radiologists should be aware of this dangerous complication particularly in patients with preexisting cardiopulmonary disease.

## 1. Introduction

In the United States, there are currently more than 500,000 patients who undergo hemodialysis as treatment for end-stage renal disease [1]. These patients require permanent access to circulation in order to perform adequate dialysis, typically either through native arteriovenous fistulas or synthetic grafts. Unfortunately, the preservation of a well-functioning dialysis access is a difficult problem in chronic dialysis patients. Over time, vascular accesses are associated with complications such as stenosis, thrombosis, infection, and aneurysm formation [2]. Hemodialysis vascular access failure is a dominant cause of morbidity and a major cost of care for end-stage renal disease patients [3]. It leads to multiple hospital readmissions and expensive radiological and surgical interventions. According to the Centers for Medicare and Medicaid services, over $1.2 billion was spent on establishing and maintaining vascular access in previous years for chronic kidney disease patients [2].

Thrombosis of hemodialysis vascular accesses is a major cause of morbidity in end-stage renal disease patients. Thrombosed accesses can be declotted by surgical or percutaneous procedures. This paper will focus on percutaneous therapy. Percutaneous catheter-based thrombolysis is commonly used in association with angioplasty to treat thrombosed hemodialysis accesses. Although major complications of these percutaneous procedures are relatively uncommon, they can result in several potentially serious complications, including pulmonary embolism, cerebral embolism, arterial embolism, bleeding with perigraft hematoma or hemorrhage, and vein rupture. Pulmonary embolism is a well-known complication of access site thrombolysis, although symptomatic pulmonary emboli are rare, especially with advances in percutaneous procedures that have occurred through the years.

We describe a case of symptomatic pulmonary embolism which occurred during percutaneous thrombolysis of a thrombosed arteriovenous fistula with review of the literature on hemodialysis access-related thromboembolism.

## 2. Case of Symptomatic Pulmonary Embolism during Percutaneous Thrombolysis

A 25-year-old African-American male with congenital kidney disease, two failed kidney transplants, and end-stage renal disease presented to an outpatient dialysis access center for management of a thrombosed native arteriovenous fistula. He had been receiving dialysis for the past three years via a right forearm fistula. Prior to that he had a thrombosed upper arm fistula. At the time of the procedure, his medications were folic acid, prednisone, megestrol, clonidine, doxazosin, lisinopril, metoprolol, minoxidil, ranitidine, and Sensipar. He was a nonsmoker, and on the day of the procedure the patient was in his usual state of health.

The patient underwent fistula thrombectomy using tissue plasminogen activator, thromboaspiration, and angioplasty. Flow was reestablished as demonstrated by fistulogram done at time of thrombolysis. About 45 minutes into the procedure, the patient became unresponsive with bradycardia and hypotension. He developed ventricular tachycardia for which he received cardiac defibrillation twice. He was resuscitated, intubated, and transferred to our hospital for further management.

**Figure 1:**
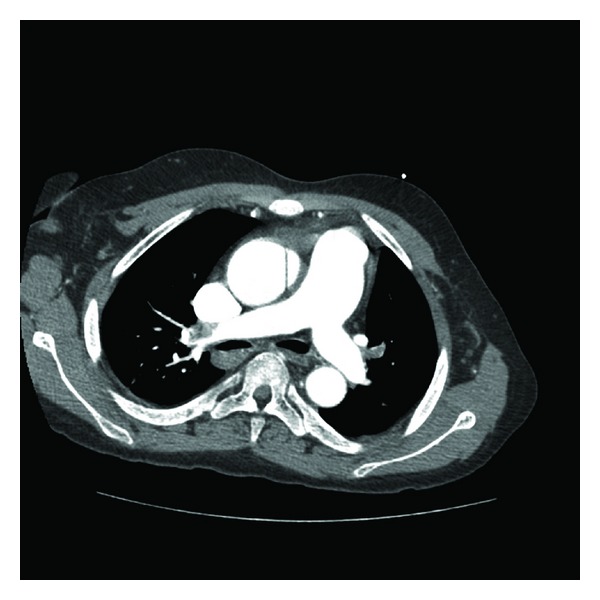
CT scan showing large filling defect in main pulmonary artery.

The patient was subsequently admitted to the cardiac care unit. He was noted to have a widened mediastinum on chest X-ray, and a CT chest with dissection protocol was done which revealed pulmonary hypertension and multiple pulmonary emboli within the bilateral lower lobes, lingula, and within the branching point of the right pulmonary trunk (Figure 1). Dopplers of bilateral lower extremities were negative for deep vein thrombosis. An echocardiogram showed a hyperdynamic left ventricle, ejection fraction of 77%, no regional wall abnormalities, moderate concentric left ventricular hypertrophy, and right ventricular elevated systolic pressure. The patient was treated for his pulmonary embolism with heparin and bridged to warfarin. He tolerated the therapy well and did not develop bleeding complications or symptoms of recurrent pulmonary emboli. He continued to receive dialysis via his now functioning right arteriovenous fistula and remained hemodynamically stable throughout the hospital stay.

## 3. Thrombosis of Hemodialysis Arteriovenous Accesses

This case illustrates a serious risk of thrombolysis of hemodialysis accesses. Hemodialysis access site thrombosis in patients on chronic hemodialysis is an important cause of morbidity in end-stage renal patients. An estimated $500 million is spent each year in the United States to create and maintain vascular access [4]. Because of the need to reestablish vascular access flow to continue hemodialysis treatments, thrombosis of a vascular access site requires prompt attention and action. Delay in restoring hemodialysis access patency can result in missed hemodialysis treatments and placement of a temporary hemodialysis catheter.

Two types of thrombi are associated with hemodialysis access thrombosis, a firm arterial plug and a soft, friable thrombus that easily disintegrates. The majority of the thrombus is composed of soft, friable thrombus [5]. Studies have shown that vascular access failure is often due to thrombosis superimposed on venous stenosis at the venous outflow of the hemodialysis access [6]. While thrombosis of arteriovenous fistulas is less frequent than thrombosis of grafts, it does pose a problem to deliverance of hemodialysis.

Thrombosis of hemodialysis access is suspected clinically. There is usually history of problems during the dialysis session, such as difficulty with cannulation, aspiration of clots, inability to achieve the target dialysis blood flow, or prolonged bleeding from needle puncture sites [7]. Abnormalities can be noted on physical exam, such as absent thrill, discontinuous bruit, or edema distal to the access site [7]. Radiographic contrast studies like fistulogram can define both arterial and venous portions of the hemodialysis access to the level of the superior vena cava. It is the definitive study prior to intervention. In addition to documenting the anatomy of the access site, the fistulogram can be used along with percutaneous angioplasty and stent placement and help with surgical revision [7].

## 4. Percutaneous Thrombolysis Procedures

Historically, the primary therapeutic option for thrombosed hemodialysis access sites involved surgery, either surgical thrombectomy or placement of a new access at a different location. These days, percutaneous options are being increasingly utilized, which can be performed by interventional radiologists or interventional nephrologists. Percutaneous thrombolysis has several advantages. It can be performed quickly following detection of thrombosis, avoids missing dialysis treatments, precludes the need for temporary catheters, and reduces the rate of hospitalization. Simultaneous angiography can detect any stenotic lesions which can be immediately treated using balloon angioplasty and/or stents [8]. Success rate of percutaneous thrombectomy has ranged between 73% and 96% [9]. While most of these studies have focused on arteriovenous grafts, percutaneous therapy of thrombosed native arteriovenous fistulas is also being increasingly utilized. Although it is more difficult to declot native fistulas than grafts, declotting of fistulas is more rewarding because it achieves better long-term results (1-year primary patency rates as high as 50% and secondary patency rates of 80%) [10]. Currently there are three types of percutaneous treatment available: pharmacological thrombolysis, pharmacomechanical thrombolysis, and mechanical thrombectomy.

Pharmacologic thrombolysis entails infusion of a thrombolytic agent (such as urokinase, streptokinase, or tissue plasminogen activator) directly into the thrombosed area. Thrombolytic therapy is administered via a multiple side-hole catheter along the length of the hemodialysis access site for 3–24 hours. Success rates of declotting using lytic drugs alone have ranged from 33% to 80% [11]. However, thrombolysis using only lytic agents is not frequently used due to prolonged treatment times, the expense of the large amount of enzyme necessary for the procedure, and the incidence of bleeding complications [12]. The current Kidney Disease Dialysis Outcomes Quality Initiative (K/DOQI) clinical practice guidelines recommend use of percutaneous thrombolysis in combination with pharmacomechanical and/or mechanical means to maximize clot clearance and reduce procedural time [7].

Pharmacomechanical thrombolysis is another option for percutaneous treatment. Formerly known as the lacing-maceration procedure, it consisted of using two catheters shaped like hockey sticks that rotated in a helical fashion while urokinase was infused into the clot [13]. Today, a popular subtype of this method is the pulse-spray technique. In this method, two catheters with multiple side-holes are inserted into the access site in a crossing fashion. A mixture of lytic agent and heparin is injected into the catheters, causing a fine, high-pressure spray to exit the catheter side-holes and fragment the thrombus [14]. Lysis-resistant clots are treated with mechanical fragmentation using a balloon catheter. Following thrombolysis, transluminal angiography and angioplasty are performed to find and treat any underlying stenotic abnormalities.

The third category of conducting percutaneous thrombolysis is mechanical thrombolysis. These include balloon declotting, thromboaspiration, pulse-spray saline thrombolysis, and thrombolysis with mechanical devices [15]. In the balloon declotting method, a balloon catheter is used for angioplasty of any stenotic area, compress the thrombus against the access wall, and push the thrombus into the central venous circulation [16]. The pulse-spray technique is similar to that used in pharmacomechanical thrombolysis, except that heparinized saline is substituted for urokinase [17]. In thromboaspiration, an angled catheter is pushed through a sheath and strong manual aspiration is created using a syringe to aspirate clots until the thrombus is cleared [18]. Several mechanical devices can liquefy and remove thrombi. These include the Arrow-Trerotola percutaneous thrombolytic device, Amplatz thrombectomy device, the Oasis hydrodynamic thrombectomy system, and the AngioJet rheolytic catheter [12].

## 5. Complications of Percutaneous Thrombolysis

Major complications of percutaneous treatment of thrombosed hemodialysis access sites are relatively uncommon. However, since percutaneous approaches are becoming increasingly utilized, awareness of these complications is important. The most common complication of thrombolytic therapy is perigraft hemorrhage which occurs in 10–33% of cases using infusion techniques. Major hemorrhagic complications requiring additional treatment can be seen in 1–7% of cases [13]. Combined pharmacomechanical techniques have greatly reduced such complications by accelerating thrombolysis and reducing lysis times. Some direct-contact mechanical devices are associated with vessel injury [11]. Less common complications include puncture site hematomas, vessel dissection or rupture, infection, and contrast reactions [11]. Arterial emboli can occur in up to 6.3% of cases regardless of the type of transcatheter approach used, although symptomatic arterial emboli are rare [12].

## 6. Pulmonary Emboli during Percutaneous Procedures

Pulmonary embolization remains a significant complication of percutaneous thrombolysis. All percutaneous thrombectomy techniques result in fragmentation of the thrombus, and these small fragments can escape from the hemodialysis access site during such procedures [19]. These fragments can then migrate through the venous circulation and embolize into segments of the pulmonary arteries [19]. It is hoped that these emboli undergo dissolution before reaching the lung or are actively undergoing dissolution if they do make it to the lung [20]. When a lytic agent is not used, there is even more concern for pulmonary emboli impacting the lung. Clinically significant pulmonary embolization during percutaneous thrombectomy procedures is an expected complication, yet it is rare, and the true incidence of pulmonary emboli is unknown. Often, if symptoms do occur, they are transient and may be attributed to conditions like congestive heart failure. Most case reports and clinical trials of pharmacomechanical and mechanical thrombolysis have reported a low incidence of pulmonary embolism, in the 0–1.0% range [12]. However, most occurrences of pulmonary emboli are clinically asymptomatic, and this rate may actually underestimate the true incidence of pulmonary emboli.

Several studies have been conducted to determine the incidence of pulmonary emboli, inclusive of clinically asymptomatic emboli. Various imaging modalities can be used to evaluate pulmonary embolism like chest CT angiography and ventilation-perfusion scans. However, studies that have evaluated the occurrence of pulmonary emboli after percutaneous thrombolysis have largely utilized ventilation-perfusion lung scans to determine the incidence of both clinically significant and insignificant (i.e., “silent”) pulmonary embolism [20–23]. These studies, for the most part, have focused at older percutaneous techniques such as pulse-spray pharmacomechanical thrombolysis or pulse-spray mechanical thrombolysis using heparinized saline. Most of the studies performed perfusion scans within 24 hours after thrombolysis procedure, but one included both prethrombolysis and immediate postprocedure lung perfusion scans [23]. The radiographic incidence of pulmonary emboli in these studies has ranged from 0 to 59% of cases. The patients in these studies experienced no symptoms suggestive of pulmonary embolization Overall, these four studies found only a few cases of symptomatic pulmonary emboli but noted that silent pulmonary emboli occurred quite frequently.

There have been a few published case reports of symptomatic pulmonary emboli after percutaneous fistula thrombectomy. Symptomatic pulmonary emboli can occur with both mechanical and percutaneous thrombolysis procedures. These events can occur with or without preexisting history of cardiovascular disease or thromboembolic events [24, 25].

The likelihood of pulmonary embolism can vary by the technique used to perform the percutaneous thrombectomy procedure, size of the thrombus, and underlying cardiopulmonary status of the patient. The technique used to declot the dialysis access can affect the occurrence of pulmonary embolization. For example, the balloon declotting method of mechanical thrombectomy involves destructing the clot and then pushing the thrombus from the access site into the central circulation. The risk of pulmonary emboli is high as one is pushing the thrombus into the central venous circulation, thereby theoretically allowing it to embolize to the pulmonary circulation. Two studies using mechanical declotting for thrombolysis found very low incidence of clinically significant pulmonary embolization [16, 26]. It is important to note, however, that neither of these studies used imaging modalities such as CT chest or ventilation-perfusion scans to determine if clinically asymptomatic pulmonary emboli were present.

 The risk from pharmacomechanical thrombolysis, such as the pulse-spray method, has a lower theoretical risk of pulmonary embolism as the thrombus is infused with a lytic agent. This method of thrombolysis is safe, quick, and reliable. Retrospective studies showed very low incidence of symptomatic pulmonary emboli with this technique [14]. However, the studies documenting safety of this technique did not use imaging to exclude presence of clinically silent pulmonary emboli. Pulse spray thrombolysis using lytic agents like urokinase is associated with lower incidence of clinically silent radiologically diagnosed pulmonary emboli as compared to heparinized saline, 18.2% with urokinase versus 64.3% with heparinized saline [22]. Findings such as these have contributed to the criticism of pulse-spray thrombolysis without lytic agents.

Percutaneous mechanical thrombectomy devices can also cause silent pulmonary emboli, although similar to the other percutaneous methods, few clinically significant pulmonary emboli have been noted. Mechanical thrombectomy generates turbulence and increases intravascular pressure which can promote migration of thrombus into the central venous circulation [27]. Several studies have been conducted comparing mechanical thrombectomy devices, such as the Amplatz thrombectomy device, with pharmacomechanical thrombolysis, such as pulse-spray thrombectomy. Review of the literature revealed no cases of clinical pulmonary embolism although no imaging was conducted to detect “silent” pulmonary emboli [16, 28].

Another factor influencing the occurrence of pulmonary embolization is size of the thrombus. In majority of cases, the volume of thrombus released during percutaneous procedures is too small to cause significant obstruction to pulmonary arterial blood flow and clinically significant pulmonary embolism. The total volume of thrombus contained within a thrombosed hemodialysis graft is often less than 5 milliliters [29]. In order to have a significant hemodynamic effect from pulmonary emboli, there would need to be occlusion of greater than 30% of the pulmonary vascular bed [12]. In arteriovenous fistulas, the volume of thrombus can be much larger due to aneurysmal dilatation, more variable anatomy, and the fact that aneurysmal segments can contain fibrotic mural thrombi resistant to thrombolysis [24].

Whether a pulmonary embolism will be clinically symptomatic is also dependent on cardiopulmonary status of the patient undergoing thrombolysis procedure. Patients with preexisting cardiopulmonary disease and inadequate cardiopulmonary reserve are likely to suffer from pulmonary emboli during thrombolysis procedure. Some patients may not be able to tolerate even a small volume of pulmonary emboli. Certain cardiopulmonary diseases like atrial fibrillation, congestive heart failure, and intrinsic lung diseases predispose to develop pulmonary embolization [21, 27]. Patients with history of multiple thrombolysis procedures in the past and previous history of thromboembolism are also predisposed to develop pulmonary emboli [24, 25]. This is of significant concern in chronic hemodialysis patients, many of whom have underlying cardiopulmonary disease and undergo frequent thrombectomy procedures. The incidence of pulmonary hypertension is also high in chronic renal dialysis patients, which can contribute to hemodynamic compromise.

## 7. Treatment

 Most cases of pulmonary embolism after percutaneous thrombolysis are subclinical and remain undiagnosed and untreated. There have been a few cases of clinically symptomatic pulmonary embolism; however it is quite uncommon and there are currently no established guidelines for the treatment of percutaneous thrombolysis-associated pulmonary embolism. Despite the lack of guidelines, most physicians tend to treat it similar to the manner in which other venous thromboembolism is treated—anticoagulation with unfractionated heparin followed by a vitamin K antagonist—although there is no supportive evidence that systemic anticoagulation offers any benefit [30]. While the National Kidney Foundation has guidelines for the management of thrombosis, no evidence is available regarding treatment to maintain graft patency after thrombosis. Studies have documented no benefit of anticoagulation in prevention of primary graft thrombosis. A study by Crowther et al. looked at the effect of low-dose warfarin (target INR of 1.4–1.9) versus placebo in patients with newly placed arteriovenous grafts [31]. The rate of graft thrombosis did not differ significantly between the two treatment groups in this study. No studies have looked at the effect of full-dose warfarin as of yet.

## 8. Conclusion

Hemodialysis patients commonly undergo percutaneous thrombectomy procedures to treat thrombosed access sites. These procedures are typically safe, cost effective, and readily available. However, subclinical pulmonary emboli do commonly occur, along with other complications. Clinicians should be alert to the possibility of clinically significant pulmonary emboli, especially in patients with preexisting cardiopulmonary disease or patients who undergo frequent thrombectomy procedures.
